# Web-based survey usefulness for studying the influence of school bags and backpacks on back and neck pain in children and adolescents - pilot study in a medium-sized town

**DOI:** 10.1186/1748-7161-8-S2-O43

**Published:** 2013-09-18

**Authors:** Wojciech Glinkowski, Izabela Czyżak, Bożena Glinkowska, Agnieszka Żukowska

**Affiliations:** 1 Chair and Department of Orthopaedics and Traumatology of Locomotor System, Center of Excellence “TeleOrto”, Baby Jesus Clinical Hospital, Medical University of Warsaw, Poland

## Background

The frequent use of websites by today’s education programs provides greater opportunities for surveying children and adolescents on health-related issues, including back pain. The prevalence of nonspecific back pain increases during adolescence [1-3]. There are several reasons for this increase in back pain in adolescents. 

## Purpose

The aim of the study was to use a web-based surveying system to investigate the possible connection between back pain in adolescents and the use of bags or backpacks. 

## Methods

The study was conducted in a town of almost 50,000 residents in north-central Poland. A cross-sectional study consisted of a web-based survey with modified questionnaire that was distributed to five schools along with an official recommendation by local authorities. 

## Results

A total of 380 questionnaires were submitted with no logical errors and highly probable answers from 220 girls (average age 13.4 years old) and 160 boys (average age 13.7 years old). The back and neck pain was mentioned by 49% of adolescents (51% of girls and 46% of boys). Thoracic and lumbar pain was mentioned by 39 girls (10%) and 100 boys (26%). The reported pain occurred intermittently (72.5%), mostly during the day (63%). Some respondents mentioned that pain occurs when they carry their bags on their shoulders (7%) or when they carry their backpacks (19%). Girls more likely carry bags (youth style), and boys prefer backpacks or rucksacks. Girls usually carry bags on the same shoulder. Of the total respondents, 65% were pain-free while being physically active 2-4 hours per week outside of the school physical education program (p<0,01). 

## Conclusions and discussion

Our study confirms the usefulness of the web-based survey for children and adolescents. In addition, we discovered a relatively high prevalence of back and neck pain in adolescents, with girls experiencing pain significantly more frequently than boys. Compared with adolescents who had no back pain, adolescents with back pain carried significantly heavier backpacks that represented a significantly greater percentage of their body weights. 
